# Environmental Lessons from China: Finding Promising Policies  in Unlikely Places

**DOI:** 10.1289/ehp.1003024

**Published:** 2011-03-10

**Authors:** Justin V. Remais, Junfeng Zhang

**Affiliations:** 1 Department of Environmental Health, Rollins School of Public Health, Emory University, Atlanta, Georgia, USA; 2 Department of Preventive Medicine, Keck School of Medicine, University of Southern California, Los Angeles, California, USA

**Keywords:** climate change, efficiency, environmental policy, pollution, renewable energy, transportation

## Abstract

Background: Alongside the major health risks posed by environmental pollution in China are recent achievements on several environmental issues that have affluent Western nations racing to catch up. The country has propelled itself to a position of leadership in clean energy and efficiency, for instance, with important consequences for public health.

Objectives: We comment on China’s challenges and recent accomplishments in addressing environmental problems from domestic pollution to global climate change. We compare China’s commitment to clean energy technology with that of other leading nations and discuss key achievements in other areas, including vehicle efficiency standards and transportation policy.

Discussion: We discuss policy directions that would secure much-needed improvements to environmental quality and health in China, along with actions that could motivate global action on issues of energy conservation and pollution reduction.

Conclusions: A comprehensive regulatory and institutional framework for environmental policy is within reach in China but will require addressing major hurdles such as the lack of an independent monitoring mechanism and the need for greater transparency and enforcement in environmental matters. Meanwhile, China can continue to set important examples by investing in renewable energy, improving energy efficiency, and limiting greenhouse gas emissions.

China is rarely a leading source for models of successful environmental management. After all, the country is known for its poor environmental quality: Air pollution levels in many of China’s cities far exceed health-based standards (Health Effects Institute 2004), and the country’s top environmental regulator classifies more than half of its water resources as too polluted for human use (Ministry of Environmental Protection 2009). Yet several recent environmental achievements and commitments have propelled the nation to an unlikely position of leadership on key environmental issues. China’s clean energy technology and pollution-reducing efficiency initiatives, for instance, have some affluent Western nations racing to catch up. Even as China wrestles with enormous environmental challenges, developed and developing country policy makers stand to learn from China’s rapid advancement in these and other areas.

Recent progress can be traced back to the extensive transformation of China’s environmental regulatory institutions over the past 20 years. In 1989, the National People’s Congress (NPC 1989) codified the Environmental Protection Law that established a legal framework that includes specific instruments for environmental management and the protection of public health. Since then, the NPC has passed dozens of laws governing resource conservation, pollution abatement, and ecological management. Although beset by lagging enforcement and compliance (Organisation for Economic Co-operation and Development 2006; Zhang 2008), momentum has increased rapidly in recent years, with rising investments aimed at improving environmental quality. Between 1991 and 2004, China steadily increased its expenditures on pollution controls, waste management, and environmental protection from 0.8% of gross domestic product (GDP) to 1.4%, although most benefits were conferred on urban areas (Shunze et al. 2007). In 2010, the NPC laid out plans for ambitious energy conservation measures, new energy technologies, and environmental protection initiatives funded by a massive outlay of $20 billion U.S. dollars (Xin and Stone 2010).

Clean energy resources are crucial to sustaining economic growth and development in China, which has raised hundreds of millions of people out of poverty (World Bank 2009) while limiting the serious environmental externalities that accompany reliance on fossil fuels. Outdoor air pollution, for instance, from vehicles, power generation, and other sources led to an estimated 470,000 premature deaths in China in 2000 (Saikawa et al. 2009; Wang and Mauzerall 2006), and highly polluting solid fuels common in rural areas has led to > 400,000 premature deaths annually from acute lower respiratory infections and other diseases (Zhang and Smith 2007). Meanwhile, China’s dependence on coal for nearly 70% of its energy supply contributes more than a quarter of global emissions of inorganic mercury (Pacyna et al. 2006), a toxicant with a range of serious health effects and an atmospheric lifetime of > 1 year, which permits its distant transport both within China and across international borders (Selin et al. 2007).

China is investing heavily to reduce its reliance on coal and other fossil fuels. In 2009, China’s investment in clean energy technology was nearly twice that of the United States ($34.6 billion vs. $18.6 billion), ranking the nation number one in investment globally (Pew Charitable Trusts 2010). This outlay raised renewable energy to about 4% of total energy use, on par with the United States. To date, China and the United States are also running neck and neck for the top two global rankings in total renewable energy production (52.5 GW vs. 53.4 GW), but China is rapidly outpacing the United States in its installed capacity, up 79% since 2005 compared with a 24% increase in the United States during the same period (Pew Charitable Trusts 2010). China has set some of the world’s most ambitious renewable energy targets, including 20 GW from solar photovoltaics, 30 GW from biomass, and a massive 150 GW from wind by 2020. These targets amount to > 15% of the country’s electricity supply (Pew Charitable Trusts 2010; Seligsohn et al. 2009; Wang et al. 2010). Investments such as these are essential for promoting innovation in clean energy, a sector with significant strategic economic and security benefits for China. These investments alone, however, will not guarantee China global leadership in the development of clean energy technology and innovation (Norris and Shenai 2010). To emerge as a leader in this and other environmental technology areas, China has much work ahead in improving higher education in science and engineering, investing in infrastructure, funding research and development, and encouraging indigenous entrepreneurship and inventive activity (Martinot and Li 2010; National Science Board 2010; Renewable Energy Policy Network for the 21st Century 2009).

The benefits of sustained investments in clean energy are many, including reduction in emissions of greenhouse gases, which is important to China because of its particular vulnerability to the adverse effects of climate change that includes increased risk of flooding, drought, and infectious disease (Campbell-Lendrum and Corvalan 2007; Gleick 2008; Xie et al. 2009; Zhou et al. 2008). Although China surpassed the United States as the world’s top carbon dioxide emitter in 2006, the country’s per capita emissions are on par with the global average, whereas the per capita emissions in the United States are approximately four times higher (Figure 1) (Boden et al. 2010). As legislation for comprehensive energy reform has stalled in the United States, China announced it would reduce its carbon intensity—the amount of carbon dioxide emitted per unit of GDP—by 40–45% by 2020 compared with 2005 (Qiu 2009). This target builds on China’s prior success: in 2005 the country set the goal to reduce energy consumption per unit GDP by 20% by 2010 (Zhou et al. 2010). The most recent data indicate that the country nearly met this ambitious target, and an early 2011 draft of the 12th Five-Year Plan proposed an additional 16% reduction by the end of 2015 (Qiu 2011). Importantly, in the global climate talks in Cancun, Mexico, in December 2010, China for the first time offered to submit its voluntary carbon emissions targets to a binding United Nations resolution, a move that was considered a major advance in global negotiations.

**Figure 1:**
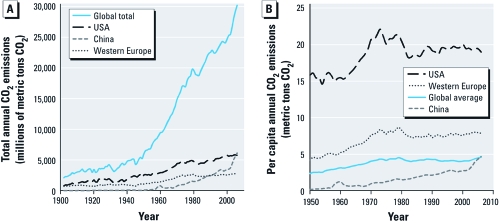
Total global, U.S., Chinese, and Western European annual carbon dioxide (CO_2_) emissions (*A*) and per capita annual CO_2_ emissions (*B*). Data from Boden et al. (2010).

Much of what China has accomplished in reducing carbon and energy intensities has come from far-reaching efficiency programs (Zhou et al. 2010). In 2004, the country was lauded for becoming the first developing economy to adopt national fuel-efficiency standards for vehicles (Oliver et al. 2009). Today, China’s standards widely exceed U.S. requirements and are the third most stringent globally (International Council on Clean Transportation 2010), behind those of Japan and the European Union (Figure 2). China has paired these standards with strict tailpipe emission controls that reduce health-damaging air pollution from vehicle traffic. Major cities, including Beijing and Hong Kong, have adopted the stringent standards set by the European Union, Euro IV, for passenger and heavy-duty vehicles, with the rest of China set to follow over the coming years (Walsh 2007).

**Figure 2:**
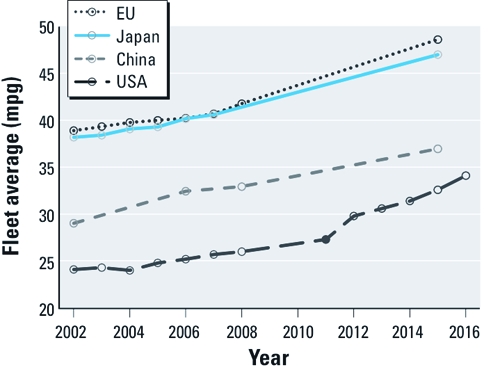
Fleet-average fuel economy (enacted targets except China 2015, which is a proposed target) for new passenger vehicles sold in select nations. Data from the International Council on Clean Transportation (2010). Mpg, miles per gallon.

Investing in clean energy and efficiency measures, on their own, will not begin to address the major environmental challenges facing China. Serious progress in improving environmental quality and health will require China to initiate major, innovative environmental policies and actions, and the country has a history of doing just that. In 1990, for instance, Hong Kong introduced a law requiring all vehicles (and power plants) to use fuel oil with a lower sulfur content, an intervention that led to sharp decreases in sulfur dioxide concentrations and an increase in life expectancy of 41 days for men and 20 days for women (Hedley et al. 2002). More recently, the remarkable removal of approximately 1.5 million cars from the roadway during the Beijing Olympic Games, in combination with the closure of high polluting industrial facilities and other actions, resulted in dramatic reductions in air pollution with both immediate and long-term health benefits (United Nations Environmental Programme 2009). Yet outdoor air pollution in Hong Kong and Beijing, among other urban centers in China, remains severe, and strategies for reducing emissions of air pollutants must be better integrated into China’s planning processes, at local and regional levels. Smart transportation planning, for instance, provides an opportunity to reduce regional air pollution and greenhouse gas emissions from vehicles, and in this area China is investing massively. The nation has constructed the world’s largest high-speed rail (HSR) network, providing clean, efficient transport on a high-speed system that, by 2012, is projected to exceed the size of the rest of the world’s HSR systems combined (International Railway Journal 2010). Yet policies promoting clean household fuels could make much larger inroads, cutting outdoor air pollution and reducing the (considerably larger) burden of disease resulting from indoor air pollution (Zhang and Smith 2007). Here, expanding China’s existing local policies banning household coal use in large cities would be effective in urban areas, and rural areas would benefit from a return to China’s history of ambitious household energy intervention programs, a history that includes the remarkable introduction of > 180 million improved stoves from the early 1980s in a program that unfortunately ended in the mid-1990s and has been followed by relatively little progress in the rural energy situation since (Sinton et al. 2004; Zhang and Smith 2007).

Despite some significant accomplishments, China routinely pursues policies that shortsightedly promote growth at the expense of environmental health (Zhang et al. 2010). In 2004, the country took a step in the right direction by officially acknowledging the economic costs of air pollution, water pollution, and industrial accidents, calculating a measure of overall economic output that discounted GDP by the costs of environmental damage and resource consumption. The resulting “green GDP” estimates, despite being highly conservative, were a wake-up call for China’s leadership: $64 billion U.S. dollars (at the official exchange rate at the time of the study in 2004) in annual costs (~ 3% of GDP) from environmental pollution and extraction paralleling the country’s rapid economic growth (State Environmental Protection Administration of China 2004). The green GDP program was short-lived, however, ending when China’s National Bureau of Statistics bowed to pressures from political and economic interests (Li and Lang 2010). Interestingly, the United States had a similar experience in the early 1990s when a green GDP program at the U.S. Bureau of Economic Analysis was dismantled after just 1 year (Matthews and Lave 2000) by a Congressional act sponsored by a representative of West Virginia’s coal country. Such economic activity measures adjusted for environmental and other factors, so-called “augmented accounts,” have long been recognized as providing better measures of final economic output than accounts limited to market transactions (Nordhaus and Kokkelenberg 1999). Despite the significant valuation and statistical challenges associated with such environmentally corrected national accounts (Alfsen et al. 2006), they are firmly grounded in mainstream economic analysis, crucial for understanding how the economy interacts with the environment, and have important implications for policy, regulatory, and business decisions (Nordhaus and Kokkelenberg 1999). For China, proper national accounting of the costs of pollution and resource consumption could increase the impact of targeted investments in environmental protection and accelerate progress across sectors. There are some worrisome signs that the country is moving in the exact opposite direction: Early in 2010 China’s National Bureau of Statistics discontinued the practice of reporting monthly coal production figures, just as the country was approaching the threshold of half of global coal consumption. A recommitment to transparent, environmentally informed national accounts could bring additional rigor, and credibility, to China’s economic and social policy making.

A comprehensive regulatory and institutional framework for environmental policy making is within reach in China (Zhang et al. 2010) but will require addressing major policy concerns such as the current role of economic growth as the single most important policy objective; the intricate relationships between levels of government that at times include the dual role of the government as a regulator and as an agent of poor environmental conditions; and the need for greater transparency and enforcement in environmental matters. Progress in these regulatory areas will grant the country greater influence on the world stage. In the meantime, even as China struggles with major environmental challenges, the country can continue to set important examples for U.S. and other Western policy makers, especially as global pollutants such as greenhouse gasses play an increasingly central role in global environmental, economic, and political debates.
